# An Improved Equilibrium Optimizer with Application in Unmanned Aerial Vehicle Path Planning

**DOI:** 10.3390/s21051814

**Published:** 2021-03-05

**Authors:** An-Di Tang, Tong Han, Huan Zhou, Lei Xie

**Affiliations:** Aeronautics Engineering College, Air Force Engineering University, Xi’an 710038, China; andisu_afeu@163.com (A.-D.T.); kgy_zhouh@163.com (H.Z.); xl310370487@163.com (L.X.)

**Keywords:** unmanned aerial vehicle, optimization algorithm, equilibrium optimizer, constrained optimization, path planning

## Abstract

The unmanned aerial vehicle (UAV) path planning problem is a type of complex multi-constraint optimization problem that requires a reasonable mathematical model and an efficient path planning algorithm. In this paper, the fitness function including fuel consumption cost, altitude cost, and threat cost is established. There are also four set constraints including maximum flight distance, minimum flight altitude, maximum turn angle, and maximum climb angle. The constrained optimization problem is transformed into an unconstrained optimization problem by using the penalty function introduced. To solve the model, a multiple population hybrid equilibrium optimizer (MHEO) is proposed. Firstly, the population is divided into three subpopulations based on fitness and different strategies are executed separately. Secondly, a Gaussian distribution estimation strategy is introduced to enhance the performance of MHEO by using the dominant information of the populations to guide the population evolution. The equilibrium pool is adjusted to enhance population diversity. Furthermore, the Lévy flight strategy and the inferior solution shift strategy are used to help the algorithm get rid of stagnation. The CEC2017 test suite was used to evaluate the performance of MHEO, and the results show that MHEO has a faster convergence speed and better convergence accuracy compared to the comparison algorithms. The path planning simulation experiments show that MHEO can steadily and efficiently plan flight paths that satisfy the constraints, proving the superiority of the MHEO algorithm while verifying the feasibility of the path planning model.

## 1. Introduction

With the continuous development of UAVs, the use of this technology in civilian commercial and military fields is increasing. Path planning and routing problems are common in the domain of commercial UAVs such as logistics drones [1,2,3]. For the defense field, its role in performing intelligence reconnaissance, combat strikes and other operations continues to be revealed. The UAV path planning is an indispensable part of the UAV mission planning. The term refers to the planning of one or more feasible, optimal, or near-optimal flight paths from the starting position to the target position which satisfies a series of constraints. Such planning also takes into account the UAV’s own performance, environmental factors and mission requirements [4]. The UAV path planning problem is a typical non-deterministic polynomial problem [5]. As the planning space and the constraints increase, the computational burden of the problem grows rapidly, and the demands on the performance of the algorithm become higher and higher. Currently, there are two types of UAV path planning methods. The first type is the traditional optimization algorithms, including the A* algorithm [6,7], the artificial potential field method [8,9], and rapidly exploring random trees [10,11]. Since traditional optimization methods rely on gradient information for a solution and are easily trapped in local optimum as the dimensionality of the problem keeps increasing [12]. Another type is the intelligent optimization algorithm, which has received a lot of attention from researchers because of its simple structure, high optimization accuracy, fast convergence and robustness.

A series of intelligent optimization algorithms have been used to solve the UAV path planning problem thanks to continuous research in its area. A hybrid genetic algorithm combined with a ray casting algorithm is proposed by Gustavo et al. [13] for solving the path planning problem of obstacle avoidance. Qu et al. [14] combine a simplified grey wolf optimizer with an improved symbiotic biological search and applies it to solving the UAV path planning problem. Yu et al. [15] present a differential evolutionary algorithm with adaptive selection variance constraints and solve the UAV path planning problem in disaster environments. Zhang and Duan [16] propose a path planning model based on the timestamp technique and solve it using an improved social class pigeon-inspired optimization. Da Silva et al. [17] combine greedy heuristics and genetic algorithms and applies them to the UAV path planning problem during emergency landings. Zhang et al. [18] integrate phase angle encoding and mutation adaptive mechanisms into the basic fruit fly optimization and applies it to plan flight path in complex 3D environments. Wu et al. [19] propose a solar-powered UAV path planning framework for complex urban environments and solve it using an improved whale optimization algorithm that includes an adaptive switching strategy and a coordinated decision mechanism. Vincent et al. [20] propose a genetic algorithm implemented in parallel on a graphics processing unit and apply it to the UAV path planning problem-solving. Puneet et al. [21] compare several existing metaheuristic optimization algorithms and propose the use of a multi-universe optimizer to obtain UAV paths with high QoS assurance.

Recently, an intelligent optimization algorithm inspired by the volume–mass balance model–the equilibrium optimizer—has been proposed. Faramarzi et al. [22] shows that EO outperforms GA [23], PSO [24], GWO [25], GSA [26], SSA [27] and covariance matrix adaptive evolution strategy (CMA-ES) [28] in terms of performance. Due to its better performance, EO has been used in a variety of practical engineering problems. Abdel-Basset et al. [29] use linear reduction diversity techniques and local minima elimination methods to improve EO performance and to estimate solar PV parameters. Agnihotri et al. [30] employ EO for solving economic dispatch problems. Improved EO based on the chaotic search is proposed in Ref. [31] and used for nonlinear planning and petrochemical applications.

Although EO has competitive advantages over other algorithms, it still suffers from slow convergence, low accuracy, and the tendency to fall into local optima in some cases. It is known from the No Free Lunch theory [32] that no optimization algorithm can solve all types of optimization problems. Therefore, the EO is improved in this paper in order to be used for solving the UAV path planning problem.

In order to improve the performance of the basic EO, this paper employs three strategies to improve the EO. Firstly, we adopt a multiple population strategy as a way to balance the exploitation and exploration ability of the algorithm. Secondly, a Gaussian distribution estimation strategy using the dominant population information to guide the evolution of individuals is introduced to enhance the algorithm performance. In addition, the Levy flight strategy and inferior solution shift strategy are used to apply perturbations to the particles to help the algorithm get rid of stagnation. The main innovations of this paper are summarized as follows.

(1) A multiple population hybrid equalization optimizer (MHEO) is proposed.

(2) A path planning model with three costs and four constraints is developed.

(3) The CEC2017 test set was used to evaluate the MHEO performance. Compared with other algorithms, MHEO has better performance.

(4) MHEO has been used to solve the path planning problem and obtains more satisfactory results.

The remainder of this paper is organized as follows. The basic EO is presented in detail in Section 2. The improvement strategy and the proposed MHEO are given in Section 3. The UAV path planning model is described in detail in Section 4. In Section 5 and Section 6, the CEC2017 function suite and the path planning problem are solved and analyzed, respectively. Finally, in Section 7, we summarize work in this paper and point out the future research that needs to be done.

## 2. The Basic EO

### 2.1. Initialization

The equilibrium optimizer (EO) is a physics-based metaheuristic algorithm. Similar to other metaheuristic algorithms, EO generates a randomly distributed initial population in the search space with the following equation.

(1) $$X_{i}=lb+r_{1}⊗(ub-lb)$$

where $X_{i}$ is the *i*^th^ generation of individual particles, $r_{1}$ is a random vector obeying a uniform distribution from 0 to 1, lb and ub are the upper and lower bounds of the search space, respectively.

### 2.2. Equilibrium Pool and Candidates

After initialization, the four particles with the best fitness are selected to form the equilibrium pool, and the equilibrium pool particles are updated after each iteration.

(2) $$X_{eq}=\{X_{eq1},X_{eq2},X_{eq3},X_{eq4},X_{eqave}\}$$

(3) $$X_{eqave}=(X_{eq1}+X_{eq2}+X_{eq3}+X_{eq4})/4$$

where $X_{eq}$ indicates the equilibrium pool, $X_{eq1}$, $X_{eq2}$, $X_{eq3}$ and $X_{eq4}$ are the four best individual particles so far, and $X_{eqave}$ is the average of the four individual particles.

### 2.3. Exponential Term

EO balances the exploitation and exploration of the algorithm by the variation of the exponential term F. The mathematical formula of F is described as follows:

(4) $$F=a_{1}\times sign(r-0.5)⊗(e^{-\lambda t}-1)$$

(5) $$t={\left(1-\frac{iter}{iter_{\max}}\right)}^{\left(a_{2}\times \frac{iter}{iter_{\max}}\right)}$$

where $a_{1}$ is a constant with a value of 2, r and λ are uniform random vectors from 0 to 1, t is a nonlinearly varying coefficient, iter denotes the number of current iterations, $iter_{\max}$ denotes the maximum number of iterations, and $a_{2}$ is a constant with a value of 1.

### 2.4. Generation Rate

The generation rate G is an important factor affecting the performance of EO and represents the exploitation ability of EO in the optimization process. The mathematical model is described as follows:

(6) $$G=GCP⊗(X_{eq}-\lambda X_{i})$$

(7) $$GCP=\{\begin{array}{cc} 0.5r_{2}, & r_{3}\geq GP\\ 0, & r_{3}<GP \end{array}$$

The GCP is a control vector that can be obtained from Equation (7). $X_{eq}$ is a randomly selected particle from the equilibrium pool. $X_{i}$ is the current particle. $r_{2}$ and $r_{3}$ are random numbers uniformly distributed from 0 to 1. GP is a constant of value 0.5.

In summary, the equation for generating candidate particles by EO can be described as follows:

(8) $$X=X_{eq}+(X-X_{eq})\cdot F+\frac{G}{\lambda v}(1-F)$$

where V is considered to be a unit volume, and the other variables are as shown above. EO balances the exploitation and exploration of the algorithm by adjusting the contribution of the second and third terms through F, where the second term helps exploration and the third term helps exploitation.

## 3. The Proposed MHEO

Compared with other metaheuristic optimization algorithms, EO solves optimization problems effectively. However, EO relies on the particles in the equilibrium pool to generate candidate particles, which still suffers from the defects of reduced population diversity and falling into the local optimum. In order to overcome the shortcomings of basic EO, this paper proposes a multiple population hybrid equilibrium optimizer (MHEO). The population is divided into three subpopulations and different position update formulas are adopted to enhance the population diversity and improve the performance of the algorithm. A stagnation perturbation strategy is introduced to help the algorithm jump out of the local optimum. The mathematical model of MHEO is described in the following detail.

### 3.1. Multipopulation Strategy

In this paper, the whole population is divided into three subpopulations according to their fitness: the exploitation population, the equilibrium population, and the exploration population. In the optimization process, the three populations are updated separately according to different strategies and then evaluated together to divide the new three subpopulations. Specifically, the exploitation population uses information from the dominant population to exploit and provide better solutions. The equilibrium population balances exploitation behavior and exploration behavior. Exploration populations are exploring more search areas and maintaining population diversity. For the optimization algorithm, extensive exploration is mainly performed in the early stage to ensure that it does not fall into the local optimum, while precise exploitation is mainly performed in the later stage to ensure convergence efficiency. Thus, the size of the three subpopulations is dynamically changing, and the mathematical model is described as follows:

(9) $$population\;size=\{\begin{array}{cc} 0.3NP, & exploitation\;population\\ 0.4NP, & equilibrium\;population\\ \left[0.3-0.3\times (\frac{iter}{iter_{\max}})\right]NP, & exploration\;population \end{array}$$

where NP is the population size. In order to avoid premature convergence, the size of exploited populations should not be large and is set to 0.3NP. Meanwhile, the exploration population will be gradually reduced as the optimization proceeds in order to ensure the convergence efficiency of the algorithm at a later stage.

In Equation (9), the three subpopulations are sorted from smallest to largest based on fitness as follows.

(10) $$P_{i}=(NP-rank(f(X_{i}))+1)/NP$$

(11) $$X_{i}=\{\begin{array}{cc} exploitation\;population, & if\;P_{i}>0.7\\ equilibrium\;population, & if\;P_{i}>\left[0.3-0.3\times (\frac{iter}{iter_{\max}})\right]\mathrm{and}\;P_{i}\leq 0.7\\ exploration\;population, & \;if\;P_{i}\leq \left[0.3-0.3\times (\frac{iter}{iter_{\max}})\right]\; \end{array}$$

### 3.2. Gaussian Distribution Estimation Strategy

The basic EO mainly uses the individuals in the equilibrium pool for updating. However, when the individuals in the equilibrium pool fall into a local optimum, the quality of the whole population will be affected. To fully utilize the information of the dominant population, the Gaussian distribution estimation strategy is introduced to enhance the performance of the algorithm considering that it can use a probability model to represent the relationship between individuals. The Gaussian distribution estimation strategy computes a probability distribution model using the current dominant population and generates new candidates based on the probability distribution model sampling. In this paper, the distribution model is estimated using a weighted maximum likelihood estimation method, and the mathematical model of this strategy is described as follows:

(12) $$X_{mean}=\sum_{i=1}^{NP/2}\omega_{i}\times X_{i}$$

(13) $$\omega_{i}=\frac{\ln(NP/2+0.5)-\ln(i)}{\sum_{i=1}^{NP/2}(\ln(NP/2+0.5)-\ln(i))}$$

(14) $$Cov\left(i\right)=\frac{1}{NP/2}{\sum_{i=1}^{NP/2}\left(X_{i}-X_{mean})(X_{i}-X_{mean}\right)}^{T}$$

where $X_{mean}$ is the weighted mean value of the dominant population, $\omega_{i}$ denotes the weighting coefficients in the dominant population in descending order of fitness, and Cov is the weighted covariance matrix of the dominant population.

For the exploitation population, we use the dominant population information to modify the direction of particle evolution as a way to enhance the optimization ability of the algorithm. The dominant population equation is as follows.

(15) $$X_{i}=Gaussian(X_{mean},Cov(i))+randn\times (X_{i}-X_{mean})$$

For the equilibrium population, $X_{eqave}$ is influenced by the four optimal individuals, which is not conducive to the algorithm to explore other regions. $X_{mean}$, however, is the information set of the dominant population and is more representative of the evolutionary direction of the population. Therefore $X_{mean}$ is used instead. The modified balance pool is described as follows:

(16) $$X_{eq}=\{X_{eq1},X_{eq2},X_{eq3},X_{eq4},X_{mean}\}$$

For the exploration population, since they are far from the optimal solution, the mean points should be far from the inferior solution as a way to drive the sampling points closer to the optimal solution, while the sampling points for each inferior solution are different, which helps to enhance the exploration ability of the algorithm. The exploration population equation is described as follows.

(17) $$X_{i}=Gaussian(X_{mean},Cov(i))+randn\times (X_{mean}-X_{i})$$

### 3.3. Stagnation Perturbation Strategy

When an algorithm stalls, two types of methods are usually used to help the algorithm jump and get rid of the stagnation. One type is the restart strategy. It helps the algorithm to get out of stagnation by randomly initializing particles in the search space. However, this method does not refer to the current population of the information and is too random to be effective in general. Therefore, this paper uses another type of method to help the algorithm get rid of stagnation–particle perturbation strategy.

#### 3.3.1. Lévy Flight Strategy

Lévy flight is an important non-Gaussian random walk whose random steps obey a large tailed probability distribution [33]. Due to the infinite and fast growth of the variance, the most important feature of this flight pattern is the ability to maximize the exploration of space in an uncertain environment. Current algorithms that apply the Lévy wandering strategy all generate random search steps centered on arbitrary individuals, and this treatment maximizes the spatial search through. However, when the step size is small, the probability of obtaining better offspring individuals around inferior solutions is small, which tends to cause computational waste and is not conducive to algorithm convergence. In MHEO, the Lévy walk performs a local search centered on the current optimal solution to balance the exploration and exploitation performance of this strategy. The specific mathematical model is described as follows:

(18) $$Step=R_{L}⊗(Xeq-R_{L}⊗X_{i})$$

(19) $$X_{i}=X_{i}+rand\cdot Step/2$$

where $R_{L}$ is a vector that obeys the Lévy distribution.

#### 3.3.2. Inferior Solution Shift Strategy

When the algorithm stalls, the shift of the mean value is considered to expand the search range as a way to enhance the population diversity. Meanwhile, it can effectively utilize the information of inferior solutions and avoid the waste of inferior solutions. The specific mathematical model is described as follows:

(20) $$X_{mean,shift}=(X_{mean}+X_{eq}+X_{i})/3$$

(21) $$X_{i}=Gaussian(X_{mean,shift},Cov(i))$$

(22) $$Cov=Cov\cdot (1-iter/iter_{\max})$$

In the optimization process, the average fitness of the dominant population is used to determine whether the algorithm is in stagnation. If the average fitness of the dominant population does not change in two consecutive iterations, the algorithm is considered to be in stagnation and the two-particle perturbation strategies above are employed. The mathematical model is described as follows:

(23) $$F_{\mathrm{mean}}=\frac{\sum_{i=1}^{0.5NP}f(X_{i})}{0.5NP}$$

(24) $$flag=\{\begin{array}{c} 1,\;if\;F_{\mathrm{mean},\mathrm{old}}\geq F_{\mathrm{mean},\mathrm{new}}\\ 0,\;if\;F_{\mathrm{mean},\mathrm{old}}<F_{\mathrm{mean},\mathrm{new}} \end{array}$$

where $F_{\mathrm{mean}}$ is the mean fitness of the dominant population. $F_{\mathrm{mean},\mathrm{old}}$ is the mean fitness of the previous generation and $F_{\mathrm{mean},\mathrm{new}}$ is the mean contemporary fitness.

After each iteration is finished, new populations are generated using the following selection mechanism.

(25) $$X_{i}(iter+1)=\{\begin{array}{c} X_{i}(iter+1),f(X_{i}(iter+1))<f(X_{i}(iter))\\ X_{i}(iter),f(X_{i}(iter+1))\geq f(X_{i}(iter)) \end{array}$$

The pseudo-code of the proposed multiple population hybrid equilibrium optimizer (MHEO) is described below (Algorithm 1).
**Algorithm 1.** The procedure of MHEO1. Set the maximum iterations as $iter_{\max}$;2. Set the population size as NP;3. Assign free parameters $a_{1}=2,a_{2}=1,GP=0.5,iter=1$;4. Initialize the position using Equation (1);5. While $iter<iter_{\max}$
6. Estimate weight mean $X_{mean}$, covariance matrix Cov using Equations (12) and (14);7. Evaluate population and form Equilibrium pool using Equation (16);8. Calculate $P_{i}$ using Equation (10);8. Generate subpopulations using Equation (11);8. Update t using Equation (5);9. For *i* = 1:*NP*10. if flag=0, 11. $X_{i}$ is updated by Equations (19) and (21);12. Else13. $if\;P_{i}>0.7$
14. $X_{i}$ is updated by Equation (15);15. Elseif $P_{i}>\left[0.3-0.3\times (\frac{iter}{iter_{\max}})\right]\mathrm{and}\;P_{i}\leq 0.7$;16. $X_{i}$ is updated by Equation (8);17. Else18. $X_{i}$ is updated by Equation (17);19. End if;20. End if;21. End if;22. Generate new populations by Equation (25)23. End for24. iter=iter+1;25. End while;26. Output the best individual $X_{eq1}$ and best fitness $f(X_{eq1})$.

## 4. UAV Path Planning Model

### 4.1. Battlefield Environment Construction

The battlefield environment construction problem is mainly to solve the establishment of 3D terrain environment and the establishment of various types of threats. In this paper, we mainly use Digital Elevation Map (DEM), and the 3D mountain terrain is described as follows.

(26) $$\Phi =\{(x,y,z)|0\leq x\leq x_{max},0\leq y\leq y_{max},0\leq z\leq z_{max}\}$$

where x, y, z are the horizontal and vertical coordinates and height of any point in the battlefield environment respectively, and $x_{max}$, $y_{max}$, $z_{max}$ are the boundary values of the battlefield environment respectively.

Since this paper mainly considers static path planning, the deployment position of enemy air defense systems and the corresponding weapon performance will be obtained by various detection methods before the mission is carried out. In addition, the UAV mainly relies on low altitude assault and defense to carry out strike missions, and when encountering enemy radar or anti-aircraft fire, it usually adopts a close ground turn for evasion instead of climbing altitude to get rid of it.

**Figure 1:**
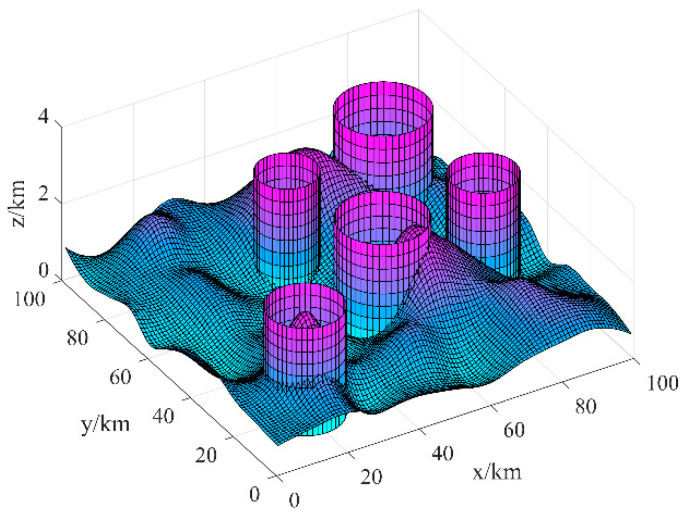
Battlefield environment.

Based on the above considerations, in order to ensure the survival rate of UAV flying to the strike target and the simplicity of the trajectory planning model, we equate the threat range of the threat source to a cylindrical obstacle model with the maximum cross-sectional area covering the threat area. In summary, the battlefield environment is constructed as shown in Figure 1.

### 4.2. Constraints and Objective Functions

Solving the path planning problem requires the establishment of a suitable fitness function and the consideration of various constraints affecting the quality of the path. The static global 3D path planning model contains two main aspects. One is the cost function, which is the objective function of the intelligent optimization algorithm. We need to consider the cost of fuel consumption, the cost of flight altitude, and the cost of the integrated threat of the UAV. The second is the performance constraints of the UAV itself, such as the maximum flight path constraint, the minimum flight altitude constraint, the maximum turn angle constraint, and the maximum climb angle constraint.

#### 4.2.1. Cost of Fuel Consumption

In actual combat missions, there is a limit to the amount of fuel a UAV can carry. The UAV cannot fly without limitation and needs to ensure that the UAV returns safely after the mission. In this paper, it is assumed that UAV performs uniform rate flight, so the size of the track length reflects the size of fuel consumption, i.e., the cost of UAV flight fuel consumption can be expressed by the track length.

(27) $${\mathrm{Cos}t}_{g}=\sum_{i=1}^{N+1}L_{i}$$

where $L_{i}$ is the length of the ith path segment.

#### 4.2.2. Cost of Flight Altitude

Flying at low altitudes is beneficial to play the role of terrain shielding and reduce the risk of being detected by radar [34]. Therefore, the UAV is required to fly at as low an altitude as possible during the mission, and the flight altitude cost can be described as follows:

(28) $${\mathrm{Cos}t}_{h}=\sum_{i=1}^{N}z_{i}$$

where $z_{i}$ is the altitude corresponding to the ith path point.

#### 4.2.3. Cost of Integrated Threat

The UAV will encounter enemy air defense systems in its mission, which include detection radar, anti-aircraft artillery, ground-to-air missiles and other threats. The above threats are approximated as a cylindrical area in the three-dimensional plane, and the detection range or strike range is used as its radius, stipulating that the UAV cannot pass through the cylindrical area.

#### 4.2.4. Constraint of Maximum Flight Distance

Assuming that the maximum range that the UAV can fly with fuel is $L_{\max}$, the maximum flight path constraint to ensure that the UAV can have enough fuel to return to base after the mission is as follows:

(29) $$2\sum_{i=1}^{N+1}L_{i}-L_{\max}<0$$

#### 4.2.5. Constraint of Minimum Flight Altitude

When the UAV performs a low-altitude surprise strike mission, although flying close to the ground can reduce the probability of being detected by radar, in the actual combat space, the terrain is complex and the risk of the aircraft crashing to the ground can easily occur. Therefore, in order to ensure UAV flight safety, a minimum flight altitude needs to be set. Each flight track altitude $H_{\min}$ flown by the UAV should satisfy the following constraint.

(30) $$H_{\min}-z_{i}<0$$

#### 4.2.6. Constraint of Maximum Turn Angle

Due to the limitation of the UAV’s own maneuverability, the maximum turn angle of the actual flight needs to be limited. At the same time, the UAV will slightly deviate from the planned trajectory during the turn. The trajectory curvature should therefore ensure that the planned path can be effectively followed by the UAV. The angle between the generated adjacent trajectory segments $\varphi_{i,j}$ cannot exceed the maximum turn angle $\varphi_{\max}$.

(31) $$\max\left|\varphi_{ij}\right|-\varphi_{\max}\leq 0$$

#### 4.2.7. Constraint of Maximum Climb Angle

The maximum climb angle is the maximum angle at which the UAV can climb or descend in flight and is another important criterion for achieving UAV maneuvers in addition to the maximum turn angle. Similar to the maximum turn angle, the climb angle between trajectory segments $\theta_{i,j}$ cannot exceed the maximum climb angle $\theta_{\max}$.

(32) $$\max\left|\theta_{ij}\right|-\theta_{\max}\leq 0$$

In summary, the objective function model for the path planning used in this paper is as follows.

(33) $$\min F=\omega_{1}\cdot \mathrm{Cos}t_{g}+\omega_{2}\cdot {\mathrm{Cos}t}_{h}+\eta \cdot Penalty$$

(34) $$Penalty=\sum_{i=1}^{n}g_{i},n=1,2,3,4$$

(35) $$g_{i}=\{\begin{array}{cc} 0, & \;\mathrm{Satisfing}\;\mathrm{constraints}\\ 1, & \mathrm{No}\;\mathrm{satisfing}\;\mathrm{constraints} \end{array}$$

where $\omega_{1}$ and $\omega_{2}$ are the proportional weight coefficients of flight fuel consumption cost and flight altitude cost respectively, and they satisfy $\omega_{1}+\omega_{2}=1$. η is the penalty function factor, and the multi-constraint optimization problem is transformed into an unconstrained optimization problem for a solution by introducing the penalty function. Algorithm 2 describes the process of using MHEO for UAV 3D flight path planning in detail.
**Algorithm 2.** MHEO for unmanned aerial vehicle (UAV) path planning1. Set the parameters of CASSA same as Algorithm 1;2. Set the start point, target point, the boundaries of battlefield space;3. Set the position and range of threats;4. While $iter<iter_{\max}$
5. Each particle represents a path; calculate the fitness F according to Equation (33) and rank the individuals according to the fitness6. Estimate weight mean $X_{mean}$, covariance matrix Cov using Equations (12) and (14);7. Evaluate population and form Equilibrium pool using Equation (16);8. Update t using Equation (5);9. For ith
$X_{i}$, if population stagnates, $X_{i}$ is updated by Equations (19) and (21);10. Else, if $X_{i}$ belongs to exploitation population, $X_{i}$ is updated by Equation (15);11. Else, if $X_{i}$ belongs to equilibrium population, $X_{i}$ is updated by Equation (8);12. Else, if $X_{i}$ belongs to exploration population, $X_{i}$ is updated by Equation (17);13. If the new path is better than before, update it;14. iter=iter+1;15. End while;16. Output the best path $X_{eq1}$ and best fitness $F(X_{eq1})$.

## 5. Experiment and Analysis of CEC 2017 Test

To comprehensively verify the superior performance of the MHEO, the algorithm is tested using the IEEE CEC2017 single-objective test function. The CEC2017 test suite includes 28 test functions, among which F1 belongs to unimodal test functions, which are used to test the convergence accuracy of the algorithm; F2–F8 belong to multimodal test functions, which are mainly used to test the ability of the algorithm to jump out of the local optimum; F9–F18 and F18–F28 are hybrid and composite functions, respectively, which are composed of complex functions and can be used to test the potential of the algorithm to solve complex optimization problems in the real world. The definition of the function and the optimal value are referred to the Ref. [35].

Three improved swarm intelligence optimization algorithms HFPSO [36], GEDGWO [37], VCS [38] and two state-of-the-art swarm intelligence optimization algorithms MPA [39], SMA [40] are used for comparison with MHEO. HFPSO is an improved particle swarm algorithm with a hybrid firefly algorithm. GEDGWO is a variant of the GWO algorithm incorporating a distribution estimation algorithm. VCS is a fusion algorithm that combines DE and CMA-ES. All these three improved algorithms have proved their good performance in the respective literature. MPA and SMA are the latest proposed better performing swarm intelligence optimization algorithms that have been used in different subjects and engineering fields in recent years. Therefore, a comparison using these algorithms can verify whether the proposed algorithms in this paper have improved in performance.

The maximum number of iterations is 600, and the population size is 500 in each experiment. The parameter settings of each algorithm refer to the original literature, as shown in Table 1. All algorithms were run independently 51 times to record the experimental results. The experimental results are shown in Appendix A, Table A1. The simulation experimental environment is AMD R7 4800U (1.80 GHz) processor and 16GB memory, and the program is run on MATLAB 2016b platform.

As shown in Table A1, MHEO performs the best on F1, which indicates the advantage of MHEO in solving the sickness function and verifies that the improvement strategy can effectively improve the exploitation ability of the algorithm. For the multimodal test functions F2–F8, MHEO achieves better solutions on six of them (F3–F7, F9), which indicates that the improvement strategy can well enhance the population diversity and avoid local optimum. HFPSO ranks first on F2. For the hybrid and composite functions F9–F28, each algorithm is superior and inferior. MHEO ranks first on 12 of the functions (F9–F13, F16–F17, F20–F22, F26–F27) and second on six of the functions (F14–F15, F18–F19, F24, F28). MPA ranks first on five of the functions tested (F14–F15, F18–F19, F24). MHEO achieves satisfactory results on F28. HFPSO and VCS obtain the best results on F25 and F23, respectively. Overall, MHEO ranked first on 19 out of 28 test functions and second on six test functions, indicating that the improvement strategy proposed in this paper can well enhance the development and exploration of the algorithm.

**Figure 2:**
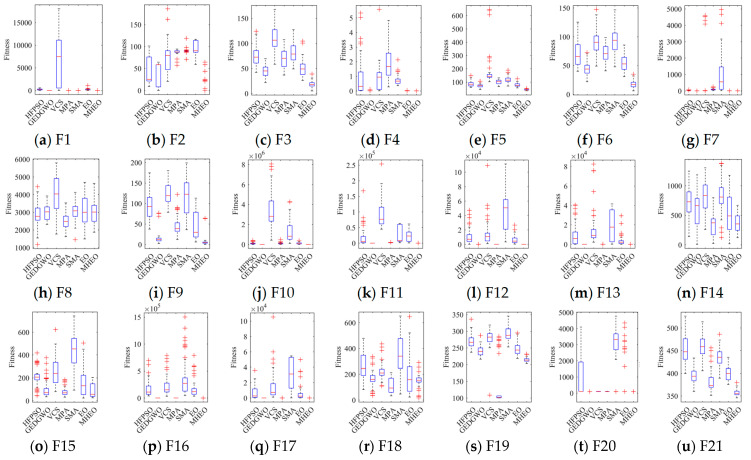

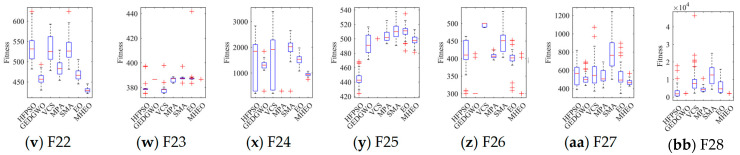
Boxes diagram of CEC2017. Subfigures (**a**-**bb**) represent functions F1–F28 respectively.

To analyze the solution quality of the improved algorithms, box plots are drawn based on the results of each algorithm solving the test function 51 times independently, as shown in Figure 2. Analysis of Figure 2 shows that the distribution of the solutions of MHEO is more concentrated in most of the test functions, which indicates that MHEO is more stable; and the median of MHEO is smaller, which indicates that MHEO solves the functions with higher accuracy.

**Figure 3:**
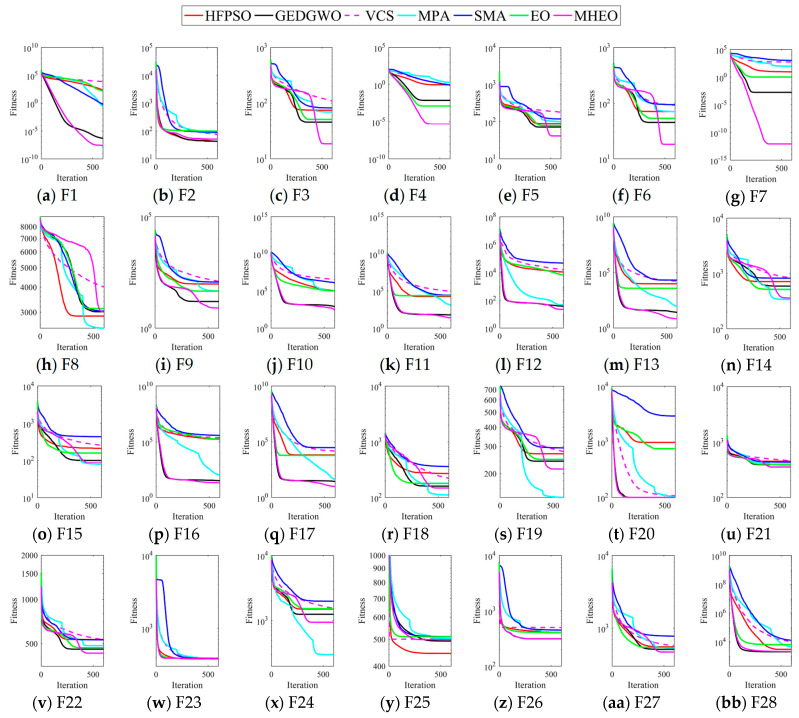
Convergence curves of CEC2017. Subfigures (**a**-**bb**) represent functions F1–F28 respectively.

Convergence speed and convergence accuracy are also important indicators of algorithm performance, and Figure 3 shows the mean error convergence curve graphs for the seven algorithms solving the CEC2017 test suite. MHEO has a faster convergence speed and better convergence accuracy in 11 tested functions (F1, F4, F7, F10–F13, F16–F17, F20, F26). For F3, F5, F6, F9, F21–F22 and F27, MHEO converges slowly at the beginning, but converges faster and has better convergence accuracy in the later stages compared to other algorithms.

In order to statistically analyze the differences between the algorithms, the Wilcoxon signed-rank test was employed to test each algorithm at a significance level of α=0.05. The results of the Wilcoxon signed-rank test are given in Appendix A, Table A2. The meanings of the symbols in Table A2. are as follows: the p-value is the probability of the observed result, and when p<0.05 which indicates a significant difference between the two algorithms. R indicates the result of the Wilcoxon signed-rank test, where “+” means that the MHEO outperforms competitor, while “-” means that MHEO is inferior to the competitor. The symbol “=” indicates that the competitors are similar to MHEO and not significantly different. We can learn from Table A2 that MHEO is significantly different from the comparison algorithm for most of the test functions. MHEO outperformed the comparison algorithm in at least 19 tested functions. It is worth noting that MHEO significantly differs from the basic EO in 26 test functions with no differences in only two test functions. Therefore, the MHEO proposed in this paper significantly outperforms the comparison algorithm in terms of performance.

Time cost is one of the important indicators of algorithm performance. Appendix A, Table A3 shows the average time cost of solving the function 51 times for each algorithm. The last row shows the average rank of the algorithm’s time cost. HFPSO ranked first, while MHEO ranked only fourth. From the theory of No Free Lunch, it is known that the increase in time cost due to the improvement of algorithm performance is acceptable. In addition, this paper studies the offline UAV path planning problem with the main consideration of accuracy, thus allowing the increase of time cost with the performance improvement.

## 6. MHEO for UAV Path Planning

In this section, we will evaluate the ability in solving the path planning problem by MHEO through simulation experiments. The experiments are conducted on a simulation platform with AMD R7 4700U, 1.8GHz,16GB RAM, and the programming environment is MATLAB R2016b. The parameters of the path planning model are set as follows: the battlefield space is $x_{\max}=100\;\mathrm{km}$, $y_{\max}=100\;\mathrm{km}$, the number of path nodes Dim = 20, the maximum turn angle is 60 degrees, and the maximum climb angle is 60 degrees. The flight fuel consumption cost $\omega_{1}=0.65$, flight altitude cost $\omega_{2}=0.35$, penalty function factor η=10,000. The number of populations is 100 and the maximum number of iterations is 500. The experiments are divided into two cases. The first one is the no-threat condition. Experiments are conducted using EO and MHEO to verify the effectiveness of the improved algorithm. The second one is with threat condition, using GEDGWO and MPA, which are ranked better in the function test, for comparison and verifying the superiority of the improved algorithm.

### 6.1. No-Threat Condition

The UAV start point is (3 km, 6 km) and the target point is (96 km, 94 km). The two algorithms were solved independently for the path planning model 20 times, and the statistical results are recorded in Table 2.

**Figure 4:**
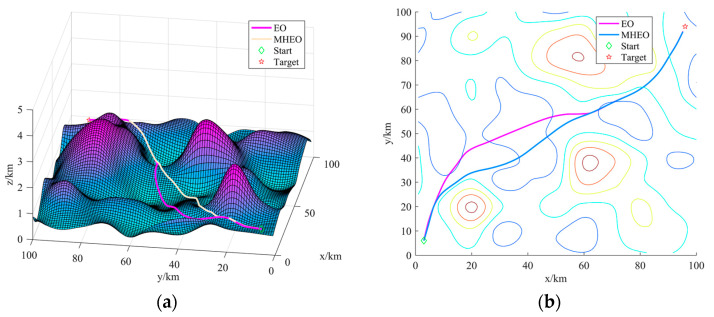
The best path of each algorithm: (**a**) path in three-dimensional space; (**b**) path in three-dimensional space.

From the statistical results in Table 2, MHEO is better than EO in terms of mean, optimal and worst values. They show the better quality of the paths planned by the MHEO proposed in this paper and indicate a greater improvement of the MHEO algorithm in convergence accuracy and algorithm robustness. Although the time cost of MHEO is larger, this paper discusses offline path planning and focuses more on the algorithm computational accuracy, so the time cost of MHEO is acceptable. Figure 4a shows the optimal 3D path for each algorithm, and Figure 4b shows the optimal path in 2D contour topography. We can intuitively see that MHEO is able to plan a shorter path in the non-threatening case, which illustrates the effectiveness of MHEO. Apart from that, we can find that the path planned by EO is the same as that of MHEO in most areas, but the path is chosen further away when passing through low-lying terrain, which leads to a bigger fitness of the path obtained by EO. On the other hand, both algorithms are able to plan paths that are close enough to the ground, which also indicates the effectiveness of the path planning model proposed in this paper.

### 6.2. Threat Condition

The UAV start point is (3 km, 6 km), the target point is (96 km, 94 km), and the threat source settings are shown in Table 3. The three algorithms are solved independently for the path planning model 20 times, and the statistical results are recorded in Table 4.

**Figure 5:**
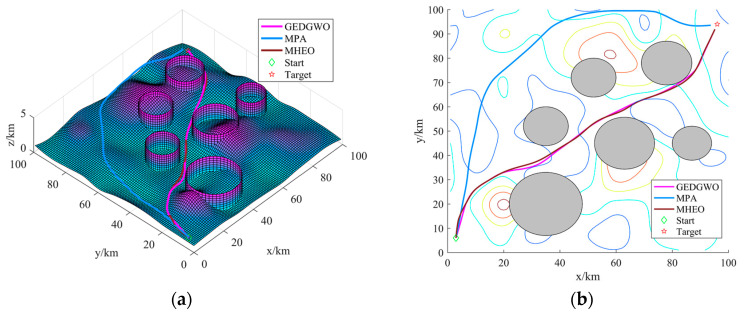
The best path of each algorithm: (**a**) path in three-dimensional space; (**b**) path in three-dimensional space.

**Figure 6:**
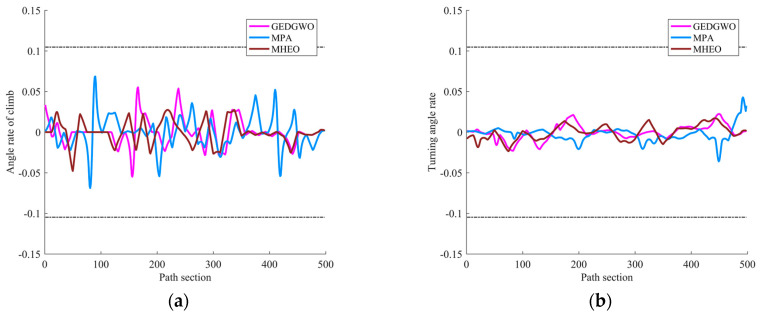
The best path of each algorithm: (**a**) climb angle; (**b**) turn angle.

**Figure 7:**
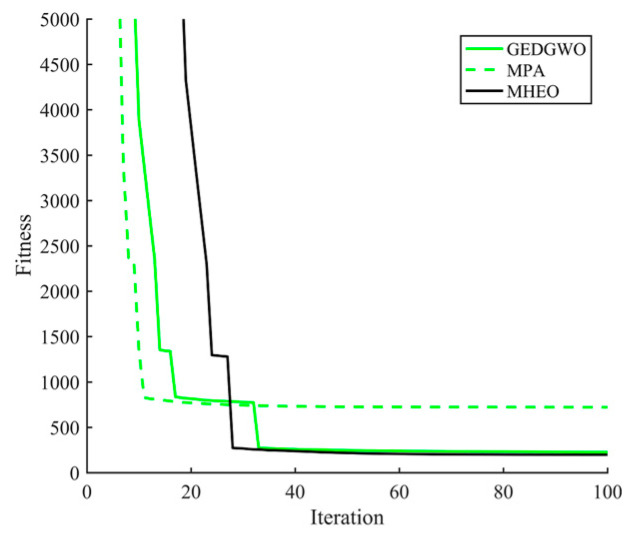
Convergence curves for three algorithms.

From the statistical results in Table 4, MHEO is better than MPA and GEDGWO in terms of mean, optimal and worst value. Meanwhile, MPA has planning failures, while MHEO can consistently plan better quality paths, indicating the better performance of the MHEO proposed in this paper. From Figure 5, we can also know that the path of MHEO can effectively avoid threats and has the shortest distance. Figure 6 gives the variation of climb angles and turn angles of the optimal paths of each algorithm. All algorithms are able to satisfy the turn angle and climb angle constraints, proving the effectiveness of this proposed trajectory planning model. Figure 7 shows the convergence curves of each algorithm. It can be seen that MHEO has higher convergence accuracy. On the other hand, the time cost of MHEO is the largest, which is the same as in the test function. Considering that this paper studies the offline path planning problem, the increased time cost of higher performance is acceptable.

## 7. Conclusions

In this paper, we proposed an improved equilibrium optimizer, a three-dimensional trajectory planning model, and the improved algorithm applied to solve the model.

In the proposed MHEO, a multiple population strategy is used to combine the basic EO and Gaussian distribution estimation strategy as a way to enhance the algorithm performance. The basic EO relies on the top three optimal individuals of the equilibrium pool for updating, which can easily lead to local optimum, while the Gaussian distribution estimation strategy is able to use the dominant population information to correct the evolutionary direction, thus avoiding this shortcoming. When the algorithm stagnates, two-particle perturbation strategies are used. A Lévy flight strategy is applied to the dominant population to help the dominant individuals get rid of the local optimum. An inferior solution shift strategy is imposed on the inferior individuals to help the worse individuals search more space.

CEC2017 was used to test the performance of the MHEO and the algorithm performance was evaluated by statistical analysis, stability analysis, convergence analysis, and Wilcoxon test. The experimental results show that MHEO has better performance and ranks first among the seven algorithms, outperforming HFPSO, GEDGWO, VCS, MPA, SMA and EO. In addition, MHEO is applied to solve the path planning model. Simulation results show that MHEO can steadily plan flight paths with better quality. The path planning model proposed in this paper is feasible. On the other hand, the time cost of MHEO is higher than that of EO due to the increased time cost caused by the calculation of the covariance matrix. However, for static path planning, the optimization accuracy is more important compared to the time cost. Therefore, the time cost due to the increased performance is acceptable.

There is still a lot of work to be done in the future. For our fund, the establishment of a single UAV path planning model and the improvement of algorithms have been completed so far. In the future, we need to further solve the multi-UAV collaborative mission planning, including multi-UAV path planning and multi-UAV target assignment. In addition, we need to further reduce the time cost of the algorithm to enable it to solve real-time problems.

## Figures and Tables

**Table 1:** Algorithm parameter setting.

Algorithm	Algorithm Parameter Setting	Algorithm	Algorithm Parameter Setting
HFPSO	$\begin{array}{l} c_{1}=c_{2}=1.49445,a=0.2,B_{0}=2\\ \gamma =1,\omega_{i}=0.9,\omega_{f}=0.5 \end{array}$	MPA	FADs=0.2,P=0.5
GEDGWO	$r_{3}\in (0,1),r_{4}\in (0,1),r_{5}\in (0,1),$	SMA	z=0.3
VCS	λ=0.5,σ=0.3	EO	$a_{1}=2,a_{2}=1$

**Table 2:** Comparison of path planning results (No-threat).

Algorithm	Mean	Best	Worst	Std	Time
EO	185.25	184.33	188.46	1.42	2.51
MHEO	184.13	182.71	186.86	1.34	2.61

**Table 3:** Threat source settings.

Threat	Type	Position/km	Radius/km	Height
Threat1	Rader	(35, 20)	13	2.8
Threat 2	missile	(35, 52)	8	2.9
Threat 3	artillery	(52, 72)	8	3.0
Threat 4	missile	(63, 45)	10.7	2.9
Threat 5	Rader	(78, 78)	9	3.1
Threat 6	artillery	(87, 45)	7	3.0

**Table 4:** Comparison of path planning results (Threat).

Algorithm	Mean	Best	Worst	Std	Time	Success
GEDGWO	217.923	184.91	248.7641	20.71729	53.92984	1
MPA	719.5117	198.3451	10228.08	2238.119	53.68744	0.95
MHEO	196.0584	184.8934	224.3117	13.22606	55.10337	1

## Data Availability

Not applicable.

## Appendix A

**Table A1:** Results statistics of seven algorithms in the CEC2017 30D test.

No		HFPSO	GEDGWO	VCS	MPA	SMA	EO	MHEO
F1	Mean	2.21 × 10^2^	4.79 × 10^−7^	7.32 × 10^3^	2.73 × 10^−1^	4.94 × 10^−1^	1.74 × 10^2^	2.58 × 10^−8^
	Std	1.35 × 10^2^	1.83 × 10^−7^	5.58 × 10^3^	3.30 × 10^−1^	3.18 × 10^−1^	1.63 × 10^2^	1.66 × 10^−8^
	Rank	6	2	7	3	4	5	1
F2	Mean	4.22 × 10^1^	4.25 × 10^1^	7.39 × 10^1^	8.68 × 10^1^	8.99 × 10^1^	9.80 × 10^1^	5.09 × 10^1^
	Std	2.87 × 10^1^	2.64 × 10^1^	3.48 × 10^1^	6.25 × 10^0^	5.12 × 10^0^	1.62 × 10^1^	1.85 × 10^1^
	Rank	1	2	4	5	6	7	3
F3	Mean	7.42 × 10^1^	4.53 × 10^1^	1.09 × 10^2^	6.89 × 10^1^	8.17 × 10^1^	5.06 × 10^1^	1.86 × 10^1^
	Std	1.82 × 10^1^	1.24 × 10^1^	2.56 × 10^1^	1.86 × 10^1^	1.99 × 10^1^	1.65 × 10^1^	6.05 × 10^0^
	Rank	5	2	7	4	6	3	1
F4	Mean	9.20 × 10^−1^	7.22 × 10^−3^	8.72 × 10^−1^	1.92 × 10^0^	7.35 × 10^−1^	1.27 × 10^−3^	4.47 × 10^−6^
	Std	1.29 × 10^0^	1.47 × 10^−2^	8.88 × 10^−1^	1.10 × 10^0^	3.21 × 10^−1^	4.09 × 10^−3^	4.39 × 10^−6^
	Rank	6	3	5	7	4	2	1
F5	Mean	8.74 × 10^1^	7.18 × 10^1^	1.79 × 10^2^	1.00 × 10^2^	1.18 × 10^2^	7.86 × 10^1^	4.13 × 10^1^
	Std	2.05 × 10^1^	1.30 × 10^1^	1.20 × 10^2^	1.68 × 10^1^	2.40 × 10^1^	1.38 × 10^1^	4.20 × 10^0^
	Rank	4	2	7	5	6	3	1
F6	Mean	7.06 × 10^1^	4.50 × 10^1^	9.19 × 10^1^	7.05 × 10^1^	9.39 × 10^1^	5.32 × 10^1^	1.82 × 10^1^
	Std	2.31 × 10^1^	1.17 × 10^1^	2.25 × 10^1^	1.44 × 10^1^	2.03 × 10^1^	1.38 × 10^1^	6.36 × 10^0^
	Rank	5	2	6	4	7	3	1
F7	Mean	8.99 × 10^0^	1.76 × 10^−3^	5.22 × 10^2^	8.87 × 10^1^	9.95 × 10^2^	1.05 × 10^0^	8.31× 10^−13^
	Std	1.11 × 10^1^	1.25 × 10^−2^	1.44 × 10^3^	4.60 × 10^1^	1.22 × 10^3^	1.19 × 10^0^	1.85× 10^−13^
	Rank	4	2	6	5	7	3	1
F8	Mean	2.87 × 10^3^	3.01 × 10^3^	4.00 × 10^3^	2.50 × 10^3^	3.04 × 10^3^	3.13 × 10^3^	3.00 × 10^3^
	Std	6.39 × 10^2^	4.30 × 10^2^	1.03 × 10^3^	4.10 × 10^2^	5.06 × 10^2^	8.08 × 10^2^	6.06 × 10^2^
	Rank	2	4	7	1	5	6	3
F9	Mean	9.43 × 10^1^	1.56 × 10^1^	1.25 × 10^2^	4.59 × 10^1^	1.16 × 10^2^	4.54 × 10^1^	8.02 × 10^0^
	Std	3.12 × 10^1^	1.54 × 10^1^	2.42 × 10^1^	2.30 × 10^1^	4.33 × 10^1^	3.21 × 10^1^	1.43 × 10^1^
	Rank	5	2	7	4	6	3	1
F10	Mean	1.04 × 10^5^	7.75 × 10^2^	3.51 × 10^6^	9.82 × 10^4^	1.31 × 10^6^	1.04 × 10^5^	2.54 × 10^2^
	Std	8.70 × 10^4^	5.82 × 10^2^	1.89 × 10^6^	7.77 × 10^4^	1.09 × 10^6^	7.47 × 10^4^	1.55 × 10^2^
	Rank	4	2	7	3	6	5	1
F11	Mean	1.83 × 10^4^	5.80 × 10^1^	9.00 × 10^4^	1.39 × 10^3^	2.71 × 10^4^	2.31 × 10^4^	2.44 × 10^1^
	Std	2.99 × 10^4^	1.90 × 10^1^	4.08 × 10^4^	3.80 × 10^2^	2.64 × 10^4^	1.84 × 10^4^	9.91 × 10^0^
	Rank	4	2	7	3	6	5	1
F12	Mean	1.05 × 10^4^	3.66 × 10^1^	1.49 × 10^4^	4.57 × 10^1^	4.71 × 10^4^	6.46 × 10^3^	2.23 × 10^1^
	Std	1.06 × 10^4^	1.32 × 10^1^	1.80 × 10^4^	1.08 × 10^1^	2.84 × 10^4^	5.98 × 10^3^	1.51 × 10^0^
	Rank	5	2	6	3	7	4	1
F13	Mean	9.39 × 10^3^	2.49 × 10^1^	1.59 × 10^4^	9.26 × 10^1^	1.99 × 10^4^	3.62 × 10^3^	6.72 × 10^0^
	Std	1.12 × 10^4^	1.56 × 10^1^	1.82 × 10^4^	2.60 × 10^1^	1.57 × 10^4^	5.19 × 10^3^	1.84 × 10^0^
	Rank	5	2	6	3	7	4	1
F14	Mean	7.09 × 10^2^	5.90 × 10^2^	8.28 × 10^2^	3.40 × 10^2^	8.18 × 10^2^	5.12 × 10^2^	3.59 × 10^2^
	Std	2.30 × 10^2^	2.89 × 10^2^	2.45 × 10^2^	1.84 × 10^2^	2.83 × 10^2^	3.04 × 10^2^	1.63 × 10^2^
	Rank	5	4	7	1	6	3	2
F15	Mean	2.11 × 10^2^	9.90 × 10^1^	2.56 × 10^2^	7.97 × 10^1^	4.34 × 10^2^	1.57 × 10^2^	8.52 × 10^1^
	Std	8.26 × 10^1^	7.71 × 10^1^	1.22 × 10^2^	3.97 × 10^1^	1.64 × 10^2^	1.24 × 10^2^	5.97 × 10^1^
	Rank	5	3	6	1	7	4	2
F16	Mean	1.67 × 10^5^	3.31 × 10^1^	2.30 × 10^5^	1.11 × 10^2^	3.75 × 10^5^	1.78 × 10^5^	2.09 × 10^1^
	Std	1.45 × 10^5^	7.76 × 10^0^	1.74 × 10^5^	2.67 × 10^1^	3.55 × 10^5^	1.70 × 10^5^	2.84 × 10^0^
	Rank	4	2	6	3	7	5	1
F17	Mean	6.60 × 10^3^	2.73 × 10^1^	1.49 × 10^4^	4.38 × 10^1^	3.00 × 10^4^	6.01 × 10^3^	9.70 × 10^0^
	Std	8.00 × 10^3^	8.80 × 10^0^	1.87 × 10^4^	8.44 × 10^0^	2.11 × 10^4^	9.98 × 10^3^	2.95 × 10^0^
	Rank	5	2	6	3	7	4	1
F18	Mean	2.70 × 10^2^	1.60 × 10^2^	2.22 × 10^2^	1.11 × 10^2^	3.59 × 10^2^	1.79 × 10^2^	1.48 × 10^2^
	Std	1.03 × 10^2^	6.87 × 10^1^	6.41 × 10^1^	5.76 × 10^1^	1.59 × 10^2^	1.30 × 10^2^	5.00 × 10^1^
	Rank	6	3	5	1	7	4	2
F19	Mean	2.69 × 10^2^	2.41 × 10^2^	2.78 × 10^2^	1.40 × 10^2^	2.93 × 10^2^	2.47 × 10^2^	2.14 × 10^2^
	Std	1.94 × 10^1^	1.40 × 10^1^	2.98 × 10^1^	7.03 × 10^1^	2.17 × 10^1^	1.72 × 10^1^	5.88 × 10^0^
	Rank	5	3	6	1	7	4	2
F20	Mean	9.77 × 10^2^	1.00 × 10^2^	1.07 × 10^2^	1.04 × 10^2^	2.90 × 10^3^	7.51 × 10^2^	1.00 × 10^2^
	Std	1.53 × 10^3^	6.36 × 10^−6^	6.10 × 10^0^	4.14 × 10^0^	1.36 × 10^3^	1.30 × 10^3^	7.02 × 10^−12^
	Rank	6	2	4	3	7	5	1
F21	Mean	4.52 × 10^2^	3.93 × 10^2^	4.62 × 10^2^	3.82 × 10^2^	4.35 × 10^2^	3.99 × 10^2^	3.57 × 10^2^
	Std	2.90 × 10^1^	1.61 × 10^1^	2.35 × 10^1^	2.39 × 10^1^	1.95 × 10^1^	1.49 × 10^1^	6.53 × 10^0^
	Rank	6	3	7	2	5	4	1
F22	Mean	5.34 × 10^2^	4.58 × 10^2^	5.32 × 10^2^	4.83 × 10^2^	5.30 × 10^2^	4.69 × 10^2^	4.30 × 10^2^
	Std	3.47 × 10^1^	1.28 × 10^1^	3.20 × 10^1^	1.79 × 10^1^	2.95 × 10^1^	1.43 × 10^1^	5.48 × 10^0^
	Rank	7	2	6	4	5	3	1
F23	Mean	3.79 × 10^2^	3.87 × 10^2^	3.78 × 10^2^	3.87 × 10^2^	3.88 × 10^2^	3.88 × 10^2^	3.87 × 10^2^
	Std	4.73 × 10^0^	1.49 × 10^−2^	3.35 × 10^0^	1.71 × 10^0^	1.69 × 10^0^	7.82 × 10^0^	1.36 × 10^−2^
	Rank	2	5	1	3	6	7	4
F24	Mean	1.50 × 10^3^	1.25 × 10^3^	1.55 × 10^3^	3.00 × 10^2^	1.98 × 10^3^	1.52 × 10^3^	9.42 × 10^2^
	Std	8.58 × 10^2^	3.15 × 10^2^	9.39 × 10^2^	5.84 × 10^−2^	3.42 × 10^2^	1.76 × 10^2^	6.97 × 10^1^
	Rank	4	3	6	1	7	5	2
F25	Mean	4.45 × 10^2^	4.92 × 10^2^	5.00 × 10^2^	5.03 × 10^2^	5.11 × 10^2^	5.10 × 10^2^	4.98 × 10^2^
	Std	1.01 × 10^1^	1.33 × 10^1^	1.51 × 10^−4^	7.53 × 10^0^	1.17 × 10^1^	9.09 × 10^0^	5.80 × 10^0^
	Rank	1	2	4	5	7	6	3
F26	Mean	4.05 × 10^2^	3.13 × 10^2^	4.96 × 10^2^	4.08 × 10^2^	4.46 × 10^2^	4.01 × 10^2^	3.11 × 10^2^
	Std	4.98 × 10^1^	3.48 × 10^1^	5.05 × 10^0^	6.99 × 10^0^	2.89 × 10^1^	2.60 × 10^1^	3.36 × 10^1^
	Rank	4	2	7	5	6	3	1
F27	Mean	5.53 × 10^2^	5.11 × 10^2^	5.69 × 10^2^	5.32 × 10^2^	7.75 × 10^2^	5.42 × 10^2^	4.68 × 10^2^
	Std	1.13 × 10^2^	6.00 × 10^1^	1.43 × 10^2^	6.92 × 10^1^	1.83 × 10^2^	1.12 × 10^2^	2.75 × 10^1^
	Rank	5	2	6	3	7	4	1
F28	Mean	2.96 × 10^3^	1.98 × 10^3^	9.38 × 10^3^	4.46 × 10^3^	1.27 × 10^4^	6.13 × 10^3^	2.03 × 10^3^
	Std	3.54 × 10^3^	2.30 × 10^1^	7.22 × 10^3^	1.42 × 10^3^	5.29 × 10^3^	3.88 × 10^3^	2.42 × 10^1^
	Rank	3	1	6	4	7	5	2
Average Rank		4.43	2.43	5.89	3.21	6.25	4.25	1.54

**Table A2:** Wilcoxon signed-rank test results (α = 0.05).

**No**	**HFPSO**				**GEDGWO**				**VCS**			
	***p*-Value**	**R+**	**R−**	**Win**	***p*-Value**	**R+**	**R−**	**Win**	***p*-Value**	**R+**	**R−**	**Win**
F1	5.15 × 10^−10^	1326	0	+	5.15 × 10^−10^	1326	0	+	5.15 × 10^−10^	1326	0	+
F2	1.44 × 10^−2^	402	924	−	4.88 × 10^−1^	589	737	=	1.26 × 10^−3^	1007	319	+
F3	5.15 × 10^−10^	1326	0	+	5.80 × 10^−10^	1324	2	+	5.15 × 10^−10^	1326	0	+
F4	5.15 × 10^−10^	1326	0	+	5.15 × 10^−10^	1326	0	+	5.15 × 10^−10^	1326	0	+
F5	5.15 × 10^−10^	1326	0	+	5.15 × 10^−10^	1326	0	+	5.15 × 10^−10^	1326	0	+
F6	5.15 × 10^−10^	1326	0	+	6.93 × 10^−10^	1321	5	+	5.15 × 10^−10^	1326	0	+
F7	5.15 × 10^−10^	1326	0	+	5.14 × 10^−10^	1326	0	−	5.15 × 10^−10^	1326	0	+
F8	3.44 × 10^−1^	562	764	=	9.78 × 10^−1^	666	660	=	9.03 × 10^−7^	1187	139	+
F9	5.15 × 10^−10^	1326	0	+	2.31 × 10^−6^	1167	159	+	5.15 × 10^−10^	1326	0	+
F10	5.15 × 10^−10^	1326	0	+	5.71 × 10^−6^	1147	179	+	5.15 × 10^−10^	1326	0	+
F11	5.15 × 10^−10^	1326	0	+	2.36 × 10^−9^	1300	26	+	5.15 × 10^−10^	1326	0	+
F12	5.15 × 10^−10^	1326	0	+	5.15 × 10^−10^	1326	0	+	5.15 × 10^−10^	1326	0	+
F13	5.15 × 10^−10^	1326	0	+	4.42 × 10^−9^	1289	37	+	5.15 × 10^−10^	1326	0	+
F14	8.18 × 10^−9^	1278	48	+	3.03 × 10^−5^	1108	218	+	1.57 × 10^−9^	1307	19	+
F15	2.72 × 10^−8^	1256	70	+	2.61 × 10^−1^	783	543	=	3.33 × 10^−9^	1294	32	+
F16	5.15 × 10^−10^	1326	0	+	5.46 × 10^−10^	1325	1	+	5.15 × 10^−10^	1326	0	+
F17	5.15 × 10^−10^	1326	0	+	9.31 × 10^−10^	1316	10	+	5.15 × 10^−10^	1326	0	+
F18	4.64 × 10^−8^	1246	80	+	1.80 × 10^−1^	806	520	=	1.01 × 10^−7^	1231	95	+
F19	5.15 × 10^−10^	1326	0	+	6.93 × 10^−10^	1321	5	+	9.66 × 10^−9^	1275	51	+
F20	5.15 × 10^−10^	1326	0	+	5.15 × 10^−10^	1326	0	−	5.15 × 10^−10^	1326	0	+
F21	5.15 × 10^−10^	1326	0	+	6.15 × 10^−10^	1323	3	+	5.15 × 10^−10^	1326	0	+
F22	5.15 × 10^−10^	1326	0	+	5.46 × 10^−10^	1325	1	+	5.15 × 10^−10^	1326	0	+
F23	4.81 × 10^−7^	126	1200	−	5.55 × 10^−1^	726	600	=	4.94 × 10^−9^	39	1287	−
F24	2.65 × 10^−6^	1164	162	+	7.13 × 10^−6^	1142	184	+	3.99 × 10^−6^	1155	171	+
F25	5.15 × 10^−10^	0	1326	−	8.92 × 10^−3^	384	942	−	2.06 × 10^−2^	910	416	+
F26	8.65 × 10^−9^	1277	49	+	5.14 × 10^−5^	1095	231	+	5.15 × 10^−10^	1326	0	+
F27	3.43 × 10^−5^	1105	221	+	1.06 × 10^−5^	1133	193	+	8.49 × 10^−6^	1138	188	+
F28	5.49 × 10^−1^	727	599	=	6.55 × 10^−9^	44	1282	−	5.15 × 10^−10^	1326	0	+
+/−/=	23/3/2				19/4/5				27/1/0			
**No**	**MPA**				**SMA**				**EO**			
	***p*−Value**	**R+**	**R−**	**Win**	***p*−Value**	**R+**	**R−**	**Win**	***p*−Value**	**R+**	**R−**	**Win**
F1	5.15 × 10^−10^	1326	0	+	5.15 × 10^−10^	1326	0	+	5.15 × 10^−10^	1326	0	+
F2	5.15 × 10^−10^	1326	0	+	5.15 × 10^−10^	1326	0	+	5.15 × 10^−10^	1326	0	+
F3	5.15 × 10^−10^	1326	0	+	5.15 × 10^−10^	1326	0	+	7.80 × 10^−10^	1319	7	+
F4	5.15 × 10^−10^	1326	0	+	5.15 × 10^−10^	1326	0	+	5.15 × 10^−10^	1326	0	+
F5	5.15 × 10^−10^	1326	0	+	5.15 × 10^−10^	1326	0	+	5.15 × 10^−10^	1326	0	+
F6	5.15 × 10^−10^	1326	0	+	5.15 × 10^−10^	1326	0	+	5.15 × 10^−10^	1326	0	+
F7	5.15 × 10^−10^	1326	0	+	5.15 × 10^−10^	1326	0	+	6.91 × 10^−9^	1281	45	+
F8	5.14 × 10^−5^	231	1095	−	7.15 × 10^−1^	702	624	=	4.53× 10^−1^	743	583	=
F9	2.36 × 10^−9^	1300	26	+	5.46 × 10^−10^	1325	1	+	3.94 × 10^−9^	1291	35	+
F10	5.15 × 10^−10^	1326	0	+	5.15 × 10^−10^	1326	0	+	5.15× 10^−10^	1326	0	+
F11	5.15 × 10^−10^	1326	0	+	5.15 × 10^−10^	1326	0	+	5.15 × 10^−10^	1326	0	+
F12	5.15 × 10^−10^	1326	0	+	5.15 × 10^−10^	1326	0	+	5.15 × 10^−10^	1326	0	+
F13	5.15 × 10^−10^	1326	0	+	5.15 × 10^−10^	1326	0	+	5.15 × 10^−10^	1326	0	+
F14	5.74 × 10^−1^	603	723	=	6.92× 10^−9^	1281	45	+	1.30 × 10^−2^	928	398	+
F15	9.48 × 10^−1^	656	670	=	6.93 × 10^−10^	1321	5	+	1.22 × 10^−3^	1008	318	+
F16	5.15 × 10^−10^	1326	0	+	5.15 × 10^−10^	1326	0	+	5.15 × 10^−10^	1326	0	+
F17	5.15 × 10^−10^	1326	0	+	5.15 × 10^−10^	1326	0	+	5.15 × 10^−10^	1326	0	+
F18	1.98 × 10^−3^	333	993	−	8.65 × 10^−9^	1277	49	+	2.45 × 10^−1^	787	539	=
F19	4.17 × 10^−8^	78	1248	−	5.15 × 10^−10^	1326	0	+	5.15 × 10^−10^	1326	0	+
F20	5.15 × 10^−10^	1326	0	+	5.15 × 10^−10^	1326	0	+	5.14 × 10^−10^	1326	0	+
F21	1.77 × 10^−9^	1305	21	+	5.15 × 10^−10^	1326	0	+	5.15 × 10^−10^	1326	0	+
F22	5.15 × 10^−10^	1326	0	+	5.15 × 10^−10^	1326	0	+	5.15 × 10^−10^	1326	0	+
F23	7.08 × 10^−1^	703	623	=	1.32 × 10^−6^	1179	147	+	6.38 × 10^−3^	954	372	+
F24	5.15 × 10^−10^	0	1326	−	7.35 × 10^−10^	1320	6	+	5.15 × 10^−10^	1326	0	+
F25	7.15 × 10^−4^	1024	302	+	7.10 × 10^−7^	1192	134	+	6.36 × 10^−8^	1240	86	+
F26	1.05 × 10^−9^	1314	12	+	5.15 × 10^−10^	1326	0	+	1.18 × 10^−9^	1312	14	+
F27	3.18 × 10^−6^	1160	166	+	6.53 × 10^−10^	1322	4	+	4.74 × 10^−5^	1097	229	+
F28	5.15 × 10^−10^	1326	0	+	5.15 × 10^−10^	1326	0	+	5.15 × 10^−10^	1326	0	+
+/−/=	21/4/3				27/0/1				26/0/2			

**Table A3:** Time cost in the CEC2017 30D test (unit: seconds).

No	HFPSO	GEDGWO	VCS	MPA	SMA	EO	MHEO
F1	0.80	1.57	1.97	3.72	20.33	2.75	2.69
F2	0.82	1.58	1.96	3.64	20.37	2.76	3.95
F3	1.08	1.82	2.29	4.16	20.60	3.01	2.98
F4	2.10	2.93	3.29	6.22	21.66	3.99	4.05
F5	1.11	1.90	2.36	4.28	20.68	3.06	2.81
F6	1.09	1.84	2.26	4.24	20.69	3.04	2.97
F7	1.14	1.83	2.29	4.36	20.81	3.05	3.06
F8	1.31	2.15	2.68	4.76	20.92	3.29	5.69
F9	0.94	1.73	2.25	3.93	20.41	2.88	3.10
F10	1.10	1.59	2.34	4.30	20.63	3.08	3.21
F11	0.98	1.44	2.26	4.08	20.58	2.90	3.28
F12	1.35	1.82	2.78	4.82	20.79	3.30	3.94
F13	0.91	1.35	2.11	3.91	20.39	2.86	3.03
F14	1.04	1.84	2.54	4.25	20.53	3.01	3.94
F15	1.82	2.69	3.39	5.75	21.42	3.80	4.56
F16	1.09	1.53	2.38	4.32	20.58	3.10	3.15
F17	6.01	6.58	7.63	14.18	25.64	7.99	8.49
F18	1.98	2.83	3.33	6.02	21.54	3.99	5.00
F19	2.49	3.44	4.05	7.17	22.07	4.50	4.54
F20	2.78	3.75	4.24	7.82	22.45	4.76	4.71
F21	3.31	4.33	7.81	8.79	22.83	5.25	5.61
F22	3.59	4.54	7.41	9.34	23.19	5.54	6.01
F23	3.25	4.39	4.53	8.66	22.79	5.20	5.90
F24	4.07	5.11	6.06	10.42	23.65	6.03	6.24
F25	4.73	5.82	8.34	11.73	24.30	6.70	8.82
F26	4.12	5.16	5.71	10.39	23.66	6.05	6.13
F27	3.13	4.19	4.81	8.51	22.67	5.15	5.84
F28	7.34	8.63	10.15	16.86	26.98	9.30	11.81
Average Rank	1.00	2.00	3.25	5.93	7.00	4.00	4.82
